# Comparison of three validated PD-L1 immunohistochemical assays in urothelial carcinoma of the bladder: interchangeability and issues related to patient selection

**DOI:** 10.3389/fimmu.2022.954910

**Published:** 2022-07-27

**Authors:** Enrico Munari, Giulia Querzoli, Matteo Brunelli, Marcella Marconi, Marco Sommaggio, Marco A. Cocchi, Guido Martignoni, George J. Netto, Anna Caliò, Linda Quatrini, Francesca R. Mariotti, Claudio Luchini, Ilaria Girolami, Albino Eccher, Diego Segala, Francesco Ciompi, Giuseppe Zamboni, Lorenzo Moretta, Giuseppe Bogina

**Affiliations:** ^1^Pathology Unit, Department of Molecular and Translational Medicine, University of Brescia, Brescia, Italy; ^2^Department of Pathology and Diagnostics, University and Hospital Trust of Verona, Verona, Italy; ^3^Pathology Unit, Department of Diagnostics and Public Health, University and Hospital Trust of Verona, Verona, Italy; ^4^Pathology Unit, IRCCS Sacro Cuore Don Calabria Hospital, Negrar di Valpolicella, Verona, Italy; ^5^Laboratory Medicine, Carlo Poma Hospital, Mantova, Italy; ^6^Pathology Unit, Pederzoli Hospital, Peschiera del Garda, Verona, Italy; ^7^Department of Pathology, University of Alabama at Birmingham, Birmingham, AL, United States; ^8^Tumor Immunology Unit, Bambino Gesù Children’s Hospital (IRCCS), Rome, Italy; ^9^Pathology Unit, Central Hospital Bolzano, Bolzano, Italy; ^10^Pathology Unit, ASST Spedali Civili, Brescia, Italy; ^11^Computational Pathology Group, Department of Pathology, Radboud University Medical Center, Nijmegen, Netherlands

**Keywords:** PD-L1, immunohistochemistry, assays, comparison, urothelial, bladder, cancer, prediction

## Abstract

Different programmed cell death-ligand 1 (PD-L1) assays and scoring algorithms are being used in the evaluation of PD-L1 expression for the selection of patients for immunotherapy in specific settings of advanced urothelial carcinoma (UC). In this paper, we sought to investigate three approved assays (Ventana SP142 and SP263, and Dako 22C3) in UC with emphasis on implications for patient selection for atezolizumab/pembrolizumab as the first line of treatment. Tumors from 124 patients with invasive UC of the bladder were analyzed using tissue microarrays (TMA). Serial sections were stained with SP263 and SP142 on Ventana Benchmark Ultra and with 22C3 on Dako Autostainer Link 48. Stains were evaluated independently by two observers and scored using the combined positive score (CPS) and tumor infiltrating immune cells (IC) algorithms. Differences in proportions (DP), overall percent agreement (OPA), positive percent agreement (PPA), negative percent agreement (NPA), and Cohen κ were calculated for all comparable cases. Good overall concordance in analytic performance was observed for 22C3 and SP263 with both scoring algorithms; specifically, the highest OPA was observed between 22C3 and SP263 (89.6%) when using CPS. On the other hand, SP142 consistently showed lower positivity rates with high differences in proportions (DP) compared with 22C3 and SP263 with both CPS and IC, and with a low PPA, especially when using the CPS algorithm. In conclusion, 22C3 and SP263 assays show comparable analytical performance while SP142 shows divergent staining results, with important implications for the selection of patients for both pembrolizumab and atezolizumab.

## Introduction

Immunotherapy with immune checkpoint inhibitors (ICI) that disrupt PD-1/PD-L1 interaction has proven highly effective in different tumor types (1), and different drugs have been approved so far by regulatory agencies for the treatment of advanced or metastatic urothelial carcinoma (UC) (2, 3). The identification of patients who may benefit the most from anti-PD-1/PD-L1 therapies is a challenging issue since a relevant percentage of patients do not respond to these treatments (4–6). The most widely used parameter for the selection of patients to be treated with immunotherapy is the immunohistochemical (IHC) evaluation of PD-L1 expression on both tumor and immune cells, given the higher response rate in patients with PD-L1 positive tumors in multiple clinical trials (7). In UC, PD-L1 expression evaluation was not considered mandatory until mid-2018, when the Food and Drug Administration (FDA) and the European Medicines Agency (EMA) recommended the use of an approved (companion) IHC PD-L1 assay as a required diagnostic for pembrolizumab and atezolizumab as the first line of therapy in cisplatin-ineligible patients. Specifically, the companion diagnostic for pembrolizumab is the 22C3 pharmDx assay evaluated using the combined positive score (CPS, defined as positive if ≥10), while for atezolizumab the companion diagnostic is Ventana SP142, evaluated with the tumor-infiltrating immune cell (IC, defined as positive if ≥5%) score. Such requirements were defined after trials demonstrated that patients receiving pembrolizumab or atezolizumab and with tumors expressing low levels of PD-L1, showed a worse survival compared with patients receiving chemotherapy (8).

Subsequently, the phase 3 KEYNOTE-361 trial, evaluating pembrolizumab as monotherapy and in combination with chemotherapy as first-line treatment for patients with advanced or metastatic UC, did not meet its primary endpoints of overall survival or progression-free survival compared with standard of care chemotherapy, regardless of PD-L1 status (9). As a result, the FDA updated the indication of pembrolizumab to be for the first-line treatment of patients with advanced or metastatic UC who are not eligible for any platinum-containing chemotherapy, without the need for PD-L1 testing.

Currently, however, the EMA still requires PD-L1 IHC evaluation for pembrolizumab as first-line monotherapy in patients who are not eligible for cisplatin-containing chemotherapy (10).

Both companion diagnostic assays for pembrolizumab and atezolizumab (22C3 and SP142, respectively) run on different dedicated instruments; however, not all laboratories can afford different platforms and the entire spectrum of companion diagnostic assays that are indicated for different drugs. In this regard, one of the most widely used assays for PD-L1 IHC evaluation is Ventana SP263, which is currently considered a companion diagnostic by the FDA for atezolizumab as adjuvant treatment in stage II-IIIA non-small cell lung cancer (NSCLC) (11). Importantly, EMA does not require a specific validated **a**ssay to be tested with a given drug.

In order to address the challenges faced by pathology laboratories, which must perform multiple PD-L1 IHC assays to screen patients who might be eligible for various ICIs, efforts are needed to harmonize the PD- L1 scoring systems for patients with urothelial carcinoma (12).

We therefore sought to investigate three approved PD-L1 assays (Ventana SP142 and SP263, and Dako 22C3) in UC with a focus on the clinical implications deriving from the use of different assays for patient selection for therapy with atezolizumab or pembrolizumab as first line of treatment.

The staining has been performed as per the manufacturer’s instruction on the appropriate platforms in diagnostic laboratories and without the involvement of industries or sponsors, in order to reflect daily diagnostic practice.

## Methods

### Patients and tumor specimens

The study cohort consisted of consecutive patients with invasive urothelial carcinoma of the bladder, who underwent the surgical resection at the IRCCS Sacro Cuore Don Calabria Hospital of Negrar di Valpolicella, Verona (Italy), between 2005 and 2015, with available slides and paraffin-embedded tissue blocks. None of the patients received therapy before surgery. Tumors were classified according to the 2016 World Health Organization classification, and staging was performed using the TNM staging manual (8th edition). Investigations have been conducted after approval by the local research ethics board according to the principles expressed in the Declaration of Helsinki.

### TMA construction

For every case, all hematoxylin and eosin–stained slides were reviewed for diagnosis confirmation by a dedicated pathologist (EM); a single block was then selected for tissue microarray (TMA) construction. For each block, 5 cores with a diameter of 1 mm were obtained from the diverse areas of the tumor. Overall, 5 TMAs were built using an automated TMA instrument.

### Immunohistochemistry and assessment of PD-L1 staining

Serial sections of the TMAs were immunohistochemically stained for PD-L1 using the standardized 22C3 pharmDx assay on the Dako Autostainer Link 48 platform (Dako, Carpinteria, Ca) and the standardized SP263 and SP142 assays on the Ventana Benchmark Ultra platform (Ventana Medical Systems, Tucson, AZ), according to manufacturer’s instructions.

The stained slides were evaluated simultaneously and blindly by a dedicated urological pathologist with expertise in PD-L1 evaluation (EM) and by a researcher after appropriate training (MC); discrepancies between the two observers were resolved by consensus. All tissue cores have been evaluated using both CPS (combined positive score) and IC (tumor infiltrating immune cells) scoring systems: CPS is defined as the as the number of positive tumor cells, lymphocytes and macrophages, divided by the total number of viable tumor cells multiplied by 100 (at least 100 viable tumor cells), while IC is defined as the area of tumor infiltrated by PD-L1-stained immune cells divided by the total tumor area, multiplied by 100% (at least 50 viable tumor cells).

Necrotic areas and staining artifacts were excluded from scoring.

### Statistical analysis

Statistical analysis was carried out using Stata. To compare the clinical performance of the assays, difference in proportions (DP), overall percent agreement (OPA), positive percent agreement (PPA), negative percent agreement (NPA), and Cohen κ were calculated for all comparable cases. The McNemar test was used to evaluate the differences in percent cell staining. A two-sided P-value < 0.05 was considered statistically significant.

## Results

### Patients and staining characteristics

The cohort consisted of 124 patients from which 620 cores were collected on TMAs; of these, 45 cores resulted to be inadequate for evaluation.

Clinicopathological data are shown in **Table 1**. All patients had invasive UC of the bladder (T1-T4), with the majority (78.2%) showing disease >pT2 and with a median age of 71 years; 77.4% were males. Lymph node status was available for 76.7% and the majority (46%) were N0. No patient received neoadjuvant therapy.

**Table 1:** Clinicopathological characteristics of patients.

Variables	N (%)
**Age (y)**	
Median (range)	71 (37-90)
**Sex**	
Male	96 (77.4)
Female	28 (22.6)
**Histology**	
Invasive bladder UC	124 (100)
**T stage**	
T1	1 (0.8)
T2	26 (21)
*T2a*	4 (3.2)
*T2b*	22 (17.8)
T3	60 (48.4)
*T3a*	29 (23.4)
*T3b*	31 (25)
T4	37 (29.8)
*T4a*	35 (28.2)
*T4b*	2 (1.6)
**N stage**	
Unknown	29 (23.3)
N0	57 (46)
*N1*	16 (13)
*N2*	22 (17.7)
*N3*	0 (0)

UC, urothelial carcinoma.

### Comparison between the 22C3 pharmDx, Ventana SP263, and Ventana SP142 standardized assays

**Figure 1:**
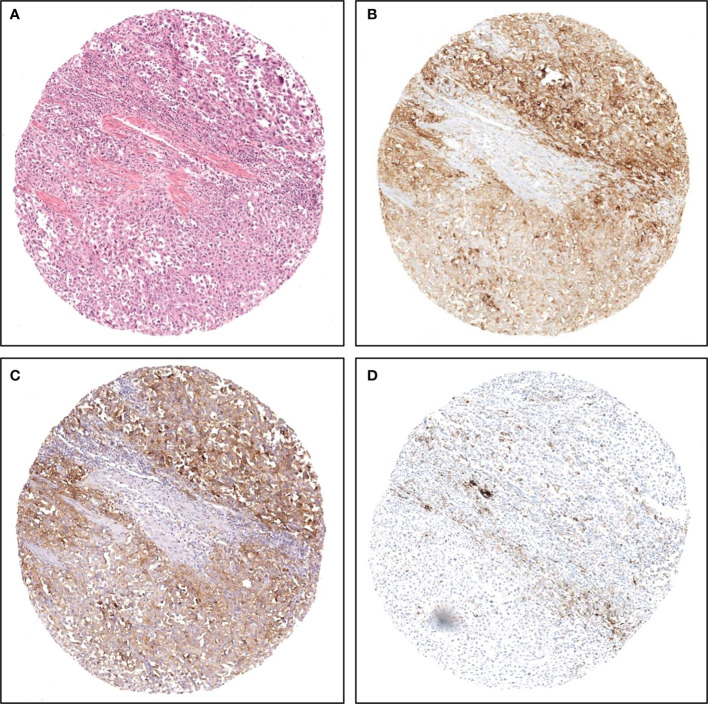
Representative images of PD-L1 staining on TMA cores from the same case. **(A)** hematoxylin and eosin; **(B)** SP263 assay; **(C)** 22C3 assay; **(D)** SP142 assay.

This analysis was performed on a per tissue core basis; the comparison was possible for 575 tissue cores. **Figure 1** shows representative IHC staining of tissue cores from the same case.

In terms of percentages of positive cores, when considering CPS (reference: 22C3, cutoff: 10), SP263 showed the highest percentage of positive cores (36.4%), while for IC (reference: SP142, cutoff: 5%), 22C3 showed the highest percentage of positive cores (**Table 2**).

For CPS, the highest OPA was observed between 22C3 and SP263 (89.6%), with a PPA and NPA of 90.2% and 89.4%, respectively. 22C3 stained fewer cases compared with SP263, with a DP of -3.6 (Cohen’s kappa 0.77). On the other hand, both 22C3 and SP263 showed lower concordance rate when compared with SP142, with an OPA of 80.7% and 76.9%, respectively. In terms of PPA, the lowest agreement was observed for SP263 *vs* SP142 (35.9%), followed by 22C3 *vs* SP142 (41.2%); Cohen’s kappa resulted to be 0.41 and 0.48, respectively.

**Table 2:** Percentages of positive cases according to assay and scoring algorithm.

	Scoring algorithm	
Clone	CPS	IC
**22C3**	32.8%	24.6%
**SP263**	36.4%	21.7%
**SP142**	13.5%	9.4%

CPS, combined positive score; IC, tumor-infiltrating immune cells.

When considering IC, compared with SP142, SP263 and 22C3 showed similar OPA (83.7% and 83.3%, respectively), with 22C3 showing a higher PPA (88.7%); the lowest DP was observed for SP263 *vs* 22C3 (-2.9) (**Table 3**).

**Table 3:** Comparison between assays 22C3, SP263, and SP142 according to different scoring algorithms.

	DP	*P* (McNemar)	κ	OPA (%)	PPA (%)	NPA (%)
**CPS**						
22C3 VS SP263	-3.6	0.00	0.77	89.6	90.2	89.4
22C3 VS SP142	19.3	0.00	0.48	80.7	41.2	99.5
SP263 VS SP142	22.9	0.00	0.41	76.9	35.9	99.7
**IC**						
SP142 VS SP263	-12.3	0.00	0.39	83.7	76.9	84.4
SP142 VS 22C3	-15.2	0.00	0.43	83.3	88.7	82.7
SP263 VS 22C3	-2.9	0.12	0.65	87.5	77.2	90.4

CPS, combined positive score; IC, tumor-infiltrating immune cells; DP, difference in proportion; OPA, overall percent agreement; PPA, positive percent agreement; NPA, negative percent agreement.

### Differences in patients selection for pembrolizumab and atezolizumab according to the assay used

This analysis was performed on a per patient basis; cases were considered positive with at least one positive core, as previously described (13).

When considered the clinically relevant cutoff for pembrolizumab (CPS≥10, reference assay: 22C3), SP263 showed a modest increase in positive cases (fold-change: 1.28), while SP142 showed an important reduction (fold-change: 0.55). When considering the clinically relevant cutoff for atezolizumab (IC≥5%, reference assay: SP142), both SP263 and 22C3 showed a significant increase in positive cases (fold change: 2.03 and 1.87, respectively) (**Table 4**).

**Figure 2:**
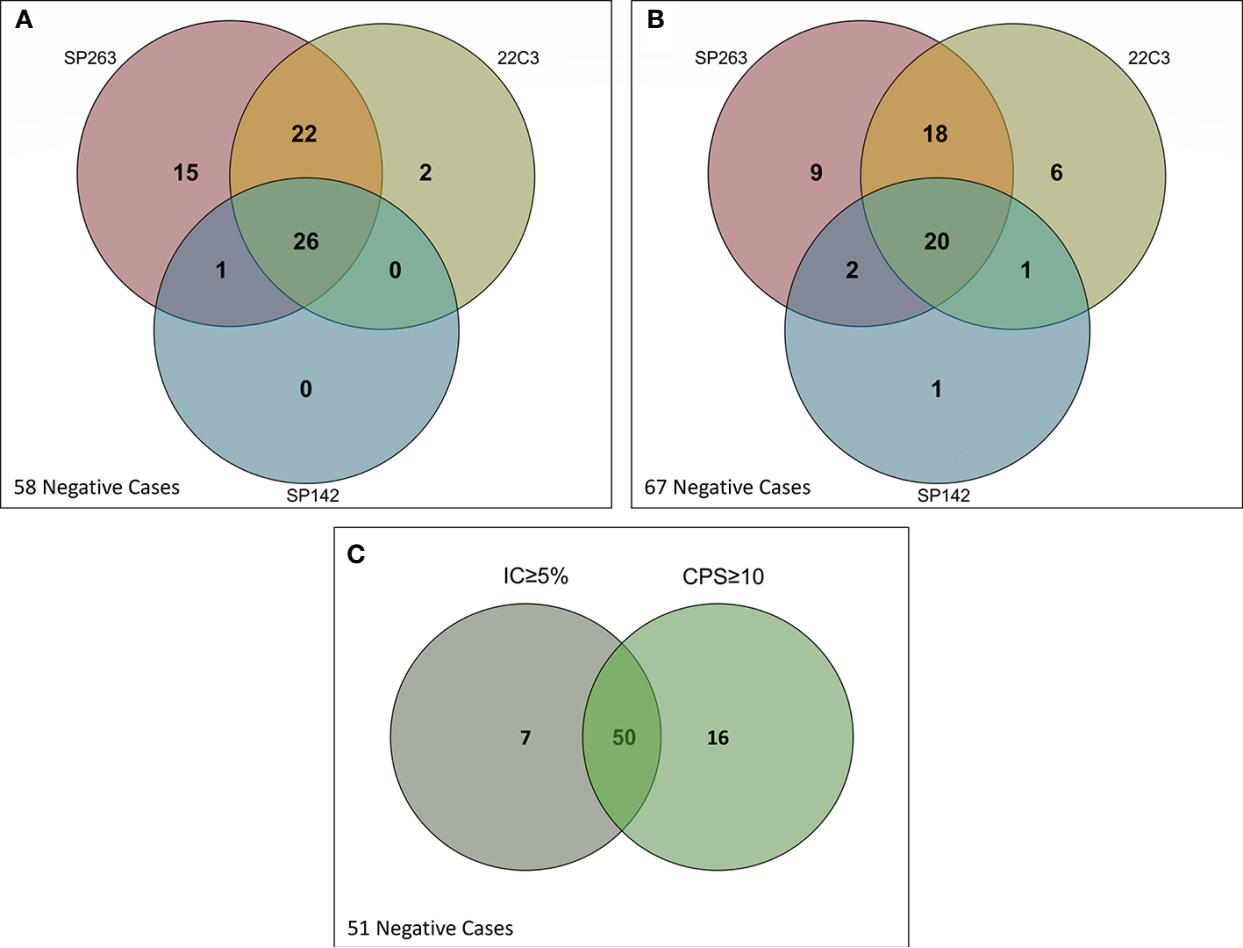
Venn diagrams showing positive and negative cases according to assays, clinical cutoffs and scoring algorithms. **(A)** CPS≥10; **(B)** IC≥5%; **(C)** CPS and IC.

In order to understand the clinical meaning in terms of patient selection according to different assays and scoring systems, we built Venn diagrams. For CPS≥10, 66 cases (53.2%) were positive with at least one assay, 26 cases (21%) were positive with all three assays, 15 cases (12%) were positive exclusively with SP263, 2 (1.6%) with 22C3 and none with SP142 (**Figure 2A**). For IC≥5%, 57 cases (46%) were positive with at least one assay, 20 (16%) were positive with all three assays, 9 (7.2%) exclusively with SP263, 6 (5%) with 22C3 and 1 (0.8%) with SP142 **(** **Figure 2B****)**. Overall, 73 cases (59%) were positive with at least one scoring system; of these, 50 cases (40.3%) were positive for both CPS≥10 and IC≥5%, 7 (5.6%) for IC≥5% only and 16 cases (13%) for CPS≥10 only **(** **Figure 2C****)**.

**Table 4:** Number of patients defined as eligible for treatment according to assay, scoring algorithm, and therapy.

PD-L1 Diagnostic Assay (Pembrolizumab)	CPS≥10	CPS<10	Fold-Change (positive cases)
22C3 (reference)	50 (40.3%)	74 (59.7%)	Reference
SP263	64 (51.6%)	60 (48.4%)	1.28
SP142	27 (22%)	97 (78%)	0.55
**PD-L1 Diagnostic Assay (Atezolizumab)**	**IC≥5%**	**IC<5%**	**Fold-Change (positive cases)**
SP142 (reference)	24 (19.4)	100 (80.6%)	Reference
SP263	49 (39.5%)	75 (60.5%)	2.03
22C3	45 (36.3%)	79 (63.7%)	1.87

CPS, combined positive score; IC, tumor-infiltrating immune cells.

### Staining heterogeneity within tissue cores

PD-L1 expression was defined as heterogeneous when one or more tissue cores within the same case showed different expression values according to the clinically relevant cutoff.

When considering CPS, heterogeneity between TMA cores from the same tumor varied according to the assay used. Percentage of discordant cases was 15.3%, 16.9% and 26.6% for SP142, 22C3 and SP263, respectively; for IC, the percentages were 17.8%, 24.2% and 29%. Such figures reflected the number of negative cases seen for each assay, which was highest for SP142 (CPS: 96 cases, IC: 99 cases), followed by 22C3 (CPS: 73 cases, IC: 77 cases) and SP263 (CPS: 61 cases, IC: 76 cases) (**Table 5**).

**Table 5:** Staining heterogeneity within tissue cores.

	Positive cases (all cores)	Negative cases (all cores)	Cases with heterogeneous cores
**SP263**			
CPS	30 (24.2%)	61 (49.2%)	33 (26.6%)
IC	12 (9.7%)	76 (61.3)	36 (29%)
**22C3**			
CPS	30 (24.2%)	73 (58.9%)	21 (16.9%)
IC	17 (13.7%)	77 (62.1%)	30 (24.2%)
**SP142**			
CPS	9 (7.3%)	96 (77.4%)	19 (15.3%)
IC	3 (2.4%)	99 (79.8%)	22 (17.8%)

CPS, combined positive score; IC, tumor-infiltrating immune cells.

### Interobserver variability

Interobserver variability between the two observers resulted to be good for CPS across all three antibodies tested, with a Cohen’s kappa of 0.91, 0.94, and 0.92 for 22C3, SP263, and SP142, respectively. A general lower concordance between observers was noted when using IC, with a Cohen’s kappa of 0.83, 0.66, and 0.78 for 22C3, SP263, and SP142, respectively (**Table 6**). In particular, a significant DP of 10.6 was observed when evaluating IC with SP263.

**Table 6:** Inter-observer agreement according to assay and scoring algorithm (Cohen's kappa).

	22C3	SP263	SP142
**CPS**	0.91	0.94	0.92
**IC**	0.83	0.66	0.78

CPS, combined positive score; IC, tumor-infiltrating immune cells.

## Discussion

PD-L1 expression evaluation is required by regulatory agencies for pembrolizumab and atezolizumab as monotherapy in patients with advanced/metastatic UC who are not eligible for cisplatin-containing chemotherapy (8, 10). Given the high cost of both validated assays and immunostaining instruments, most laboratories may rely on single tests for different indications and drugs. In this regard, the EMA does not require specific assays developed in parallel with different drugs and only recommends the use of a validated assay to evaluate PD-L1 IHC expression. Thus, potential interchangeability of assays could allow laboratories to use an assay for general PD-L1 expression evaluation, irrespective of indications.

In this work, good overall concordance in analytic performance was observed for 22C3 and SP263 with both scoring algorithms (CPS and IC); specifically, the highest OPA was observed between 22C3 and SP263 (89.6%) when using CPS. On the other hand, SP142 consistently showed low positivity rates with high differences in proportions (DP) compared with 22C3 and SP263 with both CPS and IC, and with a low positive percent agreement, especially when using the CPS algorithm.

These results are in line with those reported in a similar paper by Eckstein et al (14), who analyzed the diagnostic performance of Dako 22C3 and 28-8 and Ventana SP263 and SP142, and showed interchangeable analytical performance for 22C3, 28-8, and SP263 while SP142 displayed divergent staining results. At variance with the current data, in a prior study evaluating the concordance rates and analytical performances of SP263 and 22C3 in a cohort of non-small cell lung cancer (NSCLC) (13), we found significant differences in terms of positive percent agreement (PPA) at clinically relevant cutoffs of 1% and 50%, using the tumor proportion score algorithm (TPS). Such differences may be due at least in part to the type of scoring method used (TPS *vs* CPS/IC). However, this might not be the only explanation, since another study evaluating PD-L1 expression in head and neck squamous cell carcinoma (HNSCC) found significant discrepancies between 22C3 and SP263 using both TPS and CPS (15). On the other hand, two other recent studies evaluating SP263 and 22C3 in HNSCC also found good concordance between these assays using CPS, both on TMA and whole sections (16, 17). Therefore, besides possible differences due to scoring methods these data underline the need for multiple harmonization studies for each tumor type.

From a clinical perspective, the data herein reported can have important implications for the selection of patients for both pembrolizumab and atezolizumab. Specifically, we have shown that SP142 may select 0.55-fold fewer patients than 22C3 when considering pembrolizumab (CPS≥10), while SP263 may select 1.28-fold more patients than 22C3. On the other hand, when considering atezolizumab (IC≥5%), SP263 and 22C3 may select 2.03- and 1.87-fold more patients eligible for treatment than SP142. Furthermore, many cases are defined as positive exclusively by a specific assay. In this regard, even though there is currently no evidence of superiority for a given assay or scoring system in terms of predictive potential, such discrepancies may lead to differences in treatments for patients depending on the type of assay used to test their tumor specimens. Of note, only 40% of cases have been tested positive for both scoring systems (CPS and IC); this would lead to a significant number of patients not receiving treatment if only one drug was available between atezolizumab or pembrolizumab.

Intra-tumor heterogeneity is another well-known challenge that may hamper PD-L1 predictive value. In clinical practice, tissue can be obtained from surgical resection specimens, core needle biopsies or fine needle aspirations and for most patients with advanced disease, even in the presence of multiple metastases, only one lesion is usually sampled. In this regard, we and other have demonstrated striking topographical PD-L1 expression in non-small cell lung cancer (NSCLC), likely underpinning sub-clonal evolution (7, 18, 19). Here, we demonstrate that UC also shows important topographical heterogeneity in terms of PD-L1 expression, since a significant proportion of cases showed both positive and negative tissue cores obtained from the same tissue block, according to relevant cutoffs and scoring method.

Another important factor to consider is interobserver variability. We found almost perfect agreement when using the CPS algorithm; on the other hand, the agreement was lower when using the IC algorithm, especially with assay SP263, with a difference in proportion of 10.6. Interobserver variability is a well-known variable that can affect reliability of PD-L1 expression quantification and hamper proper patient selection (20). In fact, despite the standardization of diagnostic procedures, a number of studies have demonstrated significant variations between pathologists in the interpretation of PD-L1 staining (20, 21). Such differences, however, represent an intrinsic limitation of human visual interpretation in providing a quantitative assessment of a biomarker located in a complex tumoral and immune context (7). To this end, it would be important to implement training platform for proficiency testing to allow pathologists to test themselves on different specimen types with different assays and scoring algorithms/cutoffs (22). Moreover, more precise approaches to PD-L1 scoring, irrespective of the cell compartment, might benefit from digital pathology and artificial intelligence (23, 24). Such methods could also allow a more comprehensive evaluation of the immune contexture with the possibility to quantify tumor-infiltrating lymphocytes in light of their possible inclusion in more powerful predictive models (25, 26).

For companion diagnostic IHC, parameters such as lower limit of detection (LOD) and dynamic range are unknown to both developers and users; in this regard, Sompuram et al. demonstrated that SP142 is characterized by a very high LOD compared to other approved and laboratory-developed test (LDT) assays and therefore is less sensitive. In particular, these authors showed that SP263 and SP142 do not show overlap in their dynamic range while 22C3 shows little overlap with the analytical performance of SP142 (27). These data explain the inability to harmonize the SP142 assay with either the SP263 or the 22C3 assay. However, it must be underscored that a highly sensitive assay does not necessarily imply better prediction potential, as demonstrated in the Impassion130 study, where the benefit of atezolizumab was predominant in the SP142-positive subgroup (28).

One limitation of this study is the use of TMA instead of whole sections. However, in order to take into account the topographical heterogeneity of tumors, 5 cores were taken for each case. This has allowed us to evaluate possible PD-L1 expression discrepancies within tissue and evaluate the potential clinical impact of such discrepancies in patient selection, considering TMAs as surrogate of core tissue biopsies. Another limitation is that none of the patients of this cohort was treated with immunotherapy and, therefore, we could not draw any conclusions with regard to the actual impact of possible inter-clone discrepancies and the predictive value of PD-L1 expression according to the clinically relevant cutoffs. Finally, the evaluation of PD-L1 expression was determined by only two observers, and the final score was reached through consensus. However, inter-observer variability was not the primary aim of this study, and our results are in line with those reported in the Literature (14), underscoring the need for standardized training for pathologists evaluating PD-L1 expression.

In conclusion, 22C3 and SP263 assays show comparable analytical performance while SP142 shows divergent staining results in UC, with important implications for the selection of patients eligible for treatments with pembrolizumab and atezolizumab.

## Data availability statement

The original contributions presented in the study are included in the article/supplementary material. Further inquiries can be directed to the corresponding authors.

## Ethics statement

The studies involving human participants were reviewed and approved by the ethics committee for Clinical Research of Verona and Rovigo. Written informed consent for participation was not required for this study in accordance with the national legislation and the institutional requirements.

## Author contributions

Conceptualization: EM and GB. Data curation: EM, GB, MC, and GQ. Formal analysis: EM and GB. Funding acquisition: LM and GZ. Investigation: EM, GB, and MC. Methodology: EM, GB, MB, GM, MM, and MS. Project administration: EM and GB. Resources: LM, GB, and GZ. Software: GB. Supervision: EM and GB. Validation: AE, AC, and GN. Roles/Writing -original draft: EM and GB. Writing – review and editing: LQ, FRM, CL, IG, DS, FC, and GN. All authors contributed to the article and approved the submitted version.

## Funding

This study was partially supported by Associazione Italiana per la Ricerca sul Cancro (id 21147 and 19920). LQ and FM are recipients of Fondazione Veronesi Post-doctoral Fellowships.

## Conflict of interest

FC was Chair of the Scientific and Medical Advisory Board of TRIBVN Healthcare, France, and received advisory board fees from TRIBVN Healthcare, France in the last five years. He is shareholder of Aiosyn BV, the Netherlands.

The remaining authors declare that the research was conducted in the absence of any commercial or financial relationships that could be construed as a potential conflict of interest.

## Publisher’s note

All claims expressed in this article are solely those of the authors and do not necessarily represent those of their affiliated organizations, or those of the publisher, the editors and the reviewers. Any product that may be evaluated in this article, or claim that may be made by its manufacturer, is not guaranteed or endorsed by the publisher.
